# Chinese herbal prescriptions for osteoarthritis in Taiwan: analysis of national health insurance dataset

**DOI:** 10.1186/1472-6882-14-91

**Published:** 2014-03-07

**Authors:** Fang-Pey Chen, Ching-Mao Chang, Shinn-Jang Hwang, Yu-Chun Chen, Fun-Jou Chen

**Affiliations:** 1 Center for Traditional Medicine, Taipei Veterans General Hospital, Taipei 11217, Taiwan; 2 School of Medicine, National Yang-Ming University, Taipei 11221, Taiwan; 3 Department of Family Medicine, Taipei Veterans General Hospital, No. 201, Sec. 2, Shih-Pai Road, Taipei 11217, Beitou District, Taiwan; 4 Department of Research and Education, National Yang-Ming University Hospital, I-Lan 26042, Taiwan; 5 School of Chinese Medicine, China Medical University, Taichung 40402, Taiwan

**Keywords:** Association rule, Chinese herbal medicine, National health insurance, Osteoarthritis, Pharmaco-epidemiology, Traditional Chinese medicine

## Abstract

**Background:**

Chinese herbal medicine (CHM) has been commonly used for treating osteoarthritis in Asia for centuries. This study aimed to conduct a large-scale pharmaco-epidemiologic study and evaluate the frequency and patterns of CHM used in treating osteoarthritis in Taiwan.

**Methods:**

A complete database (total 22,520,776 beneficiaries) of traditional Chinese medicine (TCM) outpatient claims offered by the National Health Insurance program in Taiwan for the year 2002 was employed for this research. Patients with osteoarthritis were identified according to the diagnostic code of the International Classification of Disease among claimed visiting files. Corresponding prescription files were analyzed, and an association rule was applied to evaluate the co-prescription of CHM for treating osteoarthritis.

**Results:**

There were 20,059 subjects who visited TCM clinics for osteoarthritis and received a total of 32,050 CHM prescriptions. Subjects between 40 and 49 years of age comprised the largest number of those treated (19.2%), followed by 50-59 years (18.8%) and 60-69 years group (18.2%). In addition, female subjects used CHMs for osteoarthritis more frequently than male subjects (female: male = 1.89: l). There was an average of 5.2 items prescribed in the form of either an individual Chinese herb or formula in a single CHM prescription for osteoarthritis. Du-zhong (*Eucommia bark*) was the most commonly prescribed Chinese single herb, while Du-huo-ji-sheng-tang was the most commonly prescribed Chinese herbal formula for osteoarthritis. According to the association rule, the most commonly prescribed formula was Du-huo-ji-sheng-tang plus Shen-tong-zhu-yu-tang, and the most commonly prescribed triple-drug combination was Du-huo-ji-sheng-tang, Gu-sui-pu (*Drynaria fortune* (Kunze) J. Sm.), and Xu-Duan (*Himalaya teasel*). Nevertheless, further clinical trials are needed to evaluate the efficacy and safety of these CHMs for treating osteoarthritis.

**Conclusions:**

This study conducted a large scale pharmaco-epidemiology survey of Chinese herbal medicine use in OA patients by analyzing the NHIRD in Taiwan in year 2002.

## Background

Osteoarthritis (OA) is a degenerative joint disease which relates to aging, and affects the joint of hands, hips, knees, spine, and feet [1,2]. OA is the most common form of joint disease in human sparing no race or geographic area [3-5]. It affected 33.6% older people in the United States [6], and the cost of artificial knee and hip replacements for severe OA were $42.3 billion in 2009 [7]. Patients with OA suffered from swelling and pain of joints, limitation of joint motion range, limitation in walking and stair climbing, and lower quality of life [8]. The managements for OA includes weight reduction, rehabilitation, and pharmacologic therapies [9]. The most common western medicine used in treating OA is non-steroidal anti-inflammatory drugs, but some people had adverse drug reactions like gastrointestinal ulcer, bleeding and renal insufficiency [10]. OA is one of the most common musculoskeletal diseases in Taiwan. The incidence rate of OA was 37% among people aged over 50 years old in Taiwan [11].

The etiology of OA is still not fully clear, but the age, genetic, biomechanical, inflammatory, and metabolic changes of joints are the main factors [12,13]. And obesity may alter daily biomechanical exposures and make a damage effects on inflammation in OA joints [14]. Several literatures have elucidated that pro-inflammatory cytokines and anti-inflammatory cytokines like IL-1, IFN-γ, IL-6, IL-7, IL-10 and TNF-α were elevated in the OA joints [15-17]. In addition, the osteoporosis is highly associated with the prevalence of OA in menopausal women [18-20]. And the subchondral bone loss was a feature of osteoporosis and the early stage of OA [21].

Since there are many adverse side effects of conventional western medication in treating OA [22], more and more OA patients use complementary and alternative medicine (CAM) to improve the symptoms and signs of the disease joints [23]. Chinese herbal medicine [24,25], acupuncture [26], dry cupping [27], herbal patch [28] or other CAM therapies [29] were the common CAM widely accepted by OA patients.

National Health Insurance (NHI) is a universal health insurance program executed since 1995 in Taiwan which covers both western medicine and traditional Chinese Medicine (TCM) [30]. Near 98% of all inhabitants in Taiwan were covered in the NHI program at the end of 2002 [31]. However, there were no nationwide population based surveys of CHM used in treating OA so far.

## Methods

In this study, we use the complete NHI database of 2002 which covered the entire population of 22,520,776 beneficiaries in Taiwan to survey the utilization and patterns of CHM for TCM outpatients’ clinics.

### Data sources

National Health Insurance Research Dataset (NHIRD) is an electronic claim data that contains nationwide medical records of National Health Insurance (NHI) since 1995 in Taiwan. We collected the information including genders, ages, birthdays, dates of encounters, and disease diagnosis of patients from the NHIRD in the year 2002 from outpatient visiting file (CM_CD2002.DAT) and the corresponding prescription files (CM_OO2002.DAT) were also identified. And this study was assessed to the TCM outpatient visits in the NHRID was approved by the Taipei Veterans General Hospital. International Classification of Diseases, Ninth Revision, Clinical Modification (ICD-9-CM) was used to define the study population with the diagnosis of OA (ICD code: 715). Patients and institutional privacies were protected by scrambling cryptogram. Data of Chinese herbal formulae and Chinese single herbs were provided in prescription files, which could reveal the prescription and the utilization patterns.

The details of NHIRD data have been described in previous prescription patterns and the utilization analysis of CHM in different diseases like insomnia [32], constipation [33], chronic hepatitis [34], inflammatory bowel disease [35], liver cancer [36] and allergic rhinitis [37].

### Study design

Although “pattern identification as the basis for determining treatment“ is the key concept for treating patients in TCM which is different from that of Western medicine doctors, the TCM doctors in Taiwan still have to use ICD-9-CM codes to make a diagnosis for outpatient visits. Since OA has several types, the ICD-9-CM codes 715.0 (Osteoarthrosis, generalized), 715.1 (Osteoarthrosis, localized, primary), 715.2 (Osteoarthrosis, localized, secondary), 715.3 (Osteoarthrosis, localized, not specified whether primary or secondary), 715.9 (Osteoarthrosis, unspecified whether generalized or localized), 716.1 (Traumatic arthropathy) and 716.9 (Arthropathy, unspecified) were all extracted as target study subjects among the outpatients visits in this study.

### Data analysis

We used the Structure Query Language (SQL server 2008, Microsoft Corp., Redmond, WA USA) for data linkage analysis and processing. Frequency and patterns of Chinese formulae or Chinese single herbs use were taken into regular statistics by the Statistical Package for Social Science version 19.0 (SPSS Inc., Chicago, IL USA). Association rule was applied when we used International Business Machines DB2 8.1 (IBM, Armonk, NY USA) for co-prescribing prescriptions. As the support factor and confidence factor were the main determining factors, in this study, we set 0.4% as the minimum support factor and 30% as the minimum confidence level.

## Results

There were totally 6,221,426 TCM outpatients (27.6%) treated by CHM among the 22,520,776 beneficiaries under the NHI in 2002. Among these TCM outpatients, 37,163 (0.6%) patients were diagnosed with OA. In this study, we extracted 20,059 patients (54.0%) who visited the TCM outpatient clinics with the single diagnosis of OA. There were totally 32,609 CHM prescriptions for these 20,059 patients. Female patients preferred using CHM for OA more than male patients (female: male = 1.89: 1). Patients’ age between 40 and 49 years had the highest prevalence rate (19.2%), followed by age 50-59 years (18.2%), 60-69 years (18.2%) and 70-79 years (15.9%). Table 1 demonstrates the age-sex-specific frequency of CHM used in OA patients.

**Table 1:** Age-sex-specific frequency for the use of Chinese herbal medicines in patients with osteoarthritis under the national health insurance in Taiwan during 2002

**Age (years)**	**Subjects with osteoarthritis using Chinese herbal medicines**					
	**Number of patients (%)**		**Male (%)**		**Female (%)**	
<30	2,093	9.5%	916	(43.8%)	1,177	(56.2%)
30-39	2,681	13.4%	940	(35.1%)	1,741	(64.9%)
40-49	3,853	19.2%	1,274	(33.1%)	2,579	(66.9%)
50-59	3,766	18.8%	1,115	(29.6%)	2,651	(70.4%)
60-69	3,654	18.2%	1,035	(29.6%)	2,619	(70.4%)
70-79	3,191	15.9%	1,280	(40.1%)	1,911	(59.9%)
> = 80	821	4.1%	386	(47.0%)	435	(53.0%)
Total	20,059	100.0%	6,946*	(34.6%)	13,113*	(65.4%)

*male: female = 1:1.89.

The top 10 Chinese single herbs for osteoarthritis in CHM were showed in Table 2. Du-zhong (*Eucommia ulmoides*, 15.6%) was the most common used Chinese single herb, followed by Xu-duan (*Dipacus asper*, 13.8%), Niu-xi (*Achyranthes bidentata*, 11.7%), Mu-gua (*Chaenomeles lagenaria*, 10.1%), Dan-sen (*Salvia miltiorrhiza*, 9.7%), Ji-xue-teng (*Spatholobus suberectus Dunn*, 8.5%), Yan-hu-suo (*Corydalis yanhusuo*, 7.8%), Wei-ling-zian (*Clematis chinensis Osbeck*, 7.3%), Ru-xiang (*Boswellia carterii Birdw*, 5.4%), Mo-yao (*Commiphora myrrha Engl.*, 5.2%) and Gu-sui-pu (*Drynaria fortune* (Kunze) J. Sm., 4.9%).

**Table 2:** The top 10 individual Chinese herbs prescribed for osteoarthritis in Taiwan during 2002 (total prescription numbers = 32,050)

**Chinese single herb (Chinese name)**	** Generic name**	**Number of prescriptions**	**Percentage**
Du-zhong	*Eucommia ulmoides*	5,005	15.6%
Xu-duan	*Dipsacus asper*	4,419	13.8%
Niu-xi	*Achyranthes bidentata*	3,763	11.7%
Mu-gua	*Chaenomeles lagenaria*	3,240	10.1%
Dan-sen	*Salvia miltiorrhiza*	3,115	9.7%
Ji-xue-teng	Spatholobus suberectus Dunn	2,721	8.5%
Yan-hu-suo	*Corydalis yanhusuo*	2,494	7.8%
Wei-ling-xian	*Clematis chinensis Osbeck*	2,354	7.3%
Ru-xiang	*Boswellia carterii Birdw.*	1,715	5.4%
Mo-yao	*Commiphoramyrrha Engl.*	1,674	5.2%
Gu-sui-pu	*Drynaria fortune* (Kunze) J. Sm.	1,569	4.9%

Among the total 32,069 CHM prescriptions for treating OA, the top 10 Chinese herbal formulae were showed in Table 3. The most common used Chinese herbal formula was Du-huo-ji-sheng-tang (26.6%), the followed by Shu-jiang-hou-xie-tang (24.3%), Dang-gui-nian-tung-tang (11.1%), Liu-wei-di-huang-wan (8.7%), Ji-sheng-shen-qi-wan (6.6%), Chi-po-ti-huang-wan (6.5%), Kou-qi-di-huang-wan (5.9%), Xue-fu-chu-yu-tang (5.3%), Gui-zhi-shuo-yao-zhi-mu-tang (4.8%), Shou-yao-gan-tsao-tang (4.4%) and Jia-wei-xiao-yao-san (3.9%).

**Table 3:** The top 10 Chinese herbal formulae prescribed for osteoarthritis in Taiwan during 2002 (total prescription numbers = 32,050)

**Chinese herbal formulae (Chinese name)**	** Ingredients**	**Number of prescriptions (%)**
Du-huo-ji-sheng-tang	*Du-huo, Sang-ji-sheng, Ren-sen, Fu-ling, Gan-cao, Dang-guei, Shao-yao, Chuan-qiong, Di-huang, Gui-zhi, Du-zhong, Niu-xi, Xi-xin, Fang-feng, Qin-jiao*	8,538 (26.6%)
Shu-jiang-hou-xie-tang	*Dang-gue, Gan-cao, Shao-yao, Di-huang , Bai-zhu, Niu-xi, Chen-pi, Tao-ren, Wei-ling-xian, Chuan-qiong, Fang-ji, Qiang-huo, Bai-zhi, Long-dan-cao, Fu-ling, Sheng-jiang*	7,804 (24.3%)
Dang-gui-nian-tung-tang	*Qiang-huo, Yin-chen-hao, Huang-qin, Gan-cao, Zhi-mu, Zhu-ling, Ze-xie, Fang-feng, Dang-guei, Cang-zhu, Ge-gen, Ren-sen, Ku-sen, Sheng-ma, Bai-zhu*	3,560 (11.1%)
Liu-wei-di-huang-wan	*Di-huang, San-zhu-yu, Shan-yao, Mu-dan-pi, Ze-xie, Fu-ling*	2,779 (8.7%)
Ji-sheng-shen-qi-wan	*Di-huang, Shan-yao, San-zhu-yu, Ze-xie, Fu-ling, Mu-dan-pi, Rou-gui, Fu-zi, Niu-xi, Che-qian-zi*	2,126 (6.6%)
Chi-po-ti-huang-wan	*Zhi-mu, Huang-bo, Di-huang, San-zhu-yu, Shan-yao, Ze-xie, Mu-dan-pi, Fu-ling*	2,081 (6.5%)
Kou-qi-di-huang-wan	*Gou-ci, Ju-hua, Di-huang, San-zhu-yu, Shan-yao, Ze-xie, Mu-dan-pi, Fu-ling*	1,904 (5.9%)
Xue-fu-chu-yu-tang	*Dang-guei, Di-huang, Tao-ren, Hong-hua, Zhi-shi, Shao-yao, Chai-hu, Gan-cao, Jie-geng, Chuan-qiong, Niu-xi*	1,702 (5.3%)
Gui-zhi-shuo-yao-zhi-mu-tang	*Gui-zhi, Shao-yao, Bai-zhu, Zhi-mu, Ma-huang, Gan-cao, Fang-feng, Sheng-jiang, Fu-zi*	1,532 (4.8%)
Shou-yao-gan-tsao-tang	*Shao-yao, Gan-cao*	1,411 (4.4%)
Jia-wei-xiao-yao-san	*Dang-guei, Fu-ling, Zhi-zi, Bo-he, Shao-yao, Chai-hu, Gan-cao, Bai-zhu, Mu-dan-pi, Sheng-jiang*	1,249 (3.9%)

**Figure 1:**
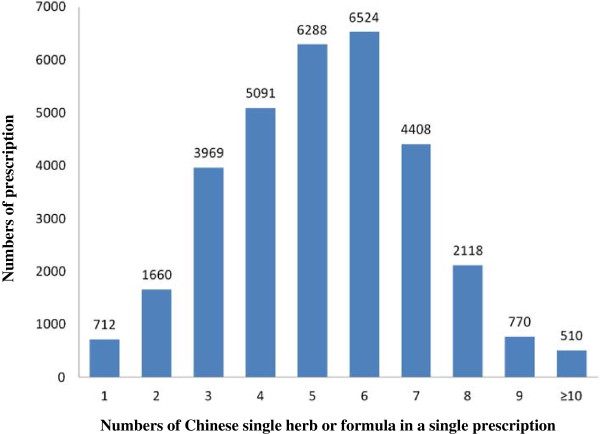
Relationship between the number of prescriptions in Taiwan in 2002 and the number of Chinese single herbs or combined ingredients of Chinese herbal formulae for subjects with osteoarthritis.

Analyzing the prescription pattern of CHM for OA, we found that a patient was given the average of 5.2 CHM in a single prescription. Six items of CHM (20.4%, Figure 1) was the most common CHM prescription with the combination of Chinese herbal formulae or Chinese single herbs, followed by 5 CHMs (19.6%) and 4 CHM combination (15.9%). We used the association rule to evaluate the co-prescription pattern of Chinese formula and Chinese single herb (Table 4 and Table 5). The most common combination of two CHM was “Shu-jiang-hou-xie-tang plus Du-huo-ji-sheng-tang”, followed by “Jia-wei-xiao-yao-san plus Du-huo-ji-sheng-tang”, and “Jia-wei-xiao-yao-san plus Shu-jing-huo-zue-tang”. The most common combination of triple CHM was “Du-huo-ji-sheng-tang, Gu-sui-pu plus Xu-duan”, followed by were “Du-huo-ji-sheng-tang, Yan-hu-suo plus Nui-xi”, “Du-huo-ji-sheng-tang, Shu-jing-huo-zue-tang plus Yan-hu-suo” and “Du-zhong, Xu-duan plus Gu-sui-pu”.

**Table 4:** The most common prescription patterns for combination Chinese herbs in a single prescription for subjects with osteoarthritis in Taiwan during 2002 (total prescription numbers = 32,050)

**Chinese herbal formulae or single herbs**	**Support (%)**	**Number of prescriptions**
*Shen-tung-chu-yu-tang, Du-huo-ji-sheng-tang*	0.7%	236
*Jia-wei-xiao-yao-san, Du-huo-ji-sheng-tang*	0.6%	197
*Jia-wei-xiao-yao-san, Shu-jing-huo-xue-tang*	0.6%	189
*Gui-zhi-shuo-yao-zhi-mu-tang, Ji-xue-teng*	0.5%	154
*Che-qian-zi, Du-huo-ji-sheng-tang*	0.5%	152
*Suan-zao-ren, Shu-jing-huo-xue-tang*	0.5%	149
*Tao-ren, Du-huo-ji-sheng-tang*	0.4%	137
*Ye-jiao-teng, Shu-jing-huo-xue-tang*	0.4%	136
*Ge-gen, Ji-ju-di-huang-wan*	0.4%	136
*Ge-gen, Xue-fu-chu-yu-tang*	0.4%	132

**Table 5:** The most common prescription patterns for the triple drug combination of Chinese herbs for subjects with osteoarthritis in Taiwan during 2002 (total prescription numbers = 32,050)

**Chinese herbal formulae or single herbs**	**Support (%)**	**Number of prescriptions**
*Du-huo-ji-sheng-tang, Gu-sui-pu, Xu-duan*	0.5%	154
*Du-huo-ji-sheng-tang, Yan-hu-suo, Niu-xi*	0.4%	133
*Du-huo-ji-sheng-tang, Shu-jing-huo-xue-tang, Yan-hu-suo*	0.4%	132
*Du-zhong, Xu-duan, Gu-sui-pu.*	0.4%	130
*Du-zhong, Wei-ling-xian, Xu-duan*	0.4%	124
*Du-huo-ji-sheng-tang, Shu-jing-huo-xue-tang, Wei-ling-xian*	0.4%	123
*Du-huo-ji-sheng-tang, Wei-ling-xian, Xu-duan*	0.4%	118
*Du-huo-ji-sheng-tang, Wei-ling-xian, Niu-xi*	0.4%	113
*Du-zhong, Xu-duan, Ji-xue-teng*	0.3%	107
*Ji-sheng-shen-qi-wan, Du-zhong, Mu-gua*	0.3%	106

## Discussion

This study is the first nationwide population based survey of the CHM use for OA patients in Taiwan. Females used CHM for OA higher than males (1.89:1), and the female aged 50-69 years had the highest prevalence of CHM use for OA. Menopause, higher percentage of obesity and osteoporosis in female may be the factors accounting for this phenomenon [38-40].

OA patients were treated with the thought of “pattern identification as the basis for determining treatment” by TCM physicians, and the prescriptions were different among TCM doctors according to their individual personal experience and their knowledge of Traditional Chinese herbs. There were few large-scale pharmaco-epidemiologic surveys of OA patients with CHM use. By analyzing the nationwide TCM outpatient clinic medical record from the NHIRD, we can evaluate the utilization and prescription patterns of CHM in treating OA.

The results showed “Du-huo-ji-sheng-tang” was the most common used Chinese herbal formula for OA. Lai et al. [41] conducted a prospective clinical study to observe 68 patients with OA knees who were treated with “Du-huo-ji-sheng-tang” for 4 weeks in 2005. They evaluated the knee condition with the Western Ontario and McMaster Universities Arthritis Index (WOMAC) [42], which is widely used in evaluating knee and hip OA with three dimensions including pain, disability and joint stiffness. They concluded that the WOMAC index scores were decreased after treatment, and the patients’ pain and stiffness in knees were released. In addition, their physical function were improved after treatment. Chen et al. [43] used “Du-huo-ji-sheng-tang” , osaminethacine and placebo in an anterior cruciate ligament transection inducing experimental OA rabbits model, and proposed that histological degeneration of cartilarge in “Du-huo-ji-sheng-tang” group was lower than the osaminethacine group and the control group. In this animal model, Du-huo-ji-sheng-tang” could inhibit chondrocytes apoptosis and could also regulate the expression of vascular endothelial growth factor (VEGF) mRNA, hypoxia-inducible factor (HIF) -1*α* mRNA.

“Shu-jiang-hou-xie-tang” was the second common Chinese herbal formula for OA in this study, and it was also the third common CHM formula for menopause in a previous literature [40]. It had been proved to have effects in treating musculoskeletal disease and connective tissue [44], and had effects of pain relief with the mechanism of increasing blood circulation in the adjuvant arthritis rats model [45]. The third common Chinese herbal formula was “Dang-gui-nian-tung-tang”, it had been shown to have anti-inflammation and immune regulation effects on experimental OA rats [46].

According to our results, “Du-zhong” was the most common used Chinese single herb in treating OA, and it was also one of the compositions in Du-huo-ji-sheng-tang. In the experiments of ovariectomy-induced rats, it could reduce postmenopausal osteoporosis, body weight, body mass index and fat tissue [47,48]. And “Du-zhong” was also the most common used Chinese single herb in treating osteoporosis in previous publication [40]. Since osteoporosis and osteoarthritis were commonly seen in menopausal and post-menopausal female, the CHM prescriptions for these two diseases were similar.

“Xu-duan" was the second most commonly used Chinese single herb for OA. It was shown to increase bone mineral density and also had osteo-protective effect in animal model [49,50]. “Xu-dan” can also improve degeneration of cartilage and bone in the OA mice model [51]. The third common one to treat OA was “Nui-xi”. It could improve bone mineral density and relieve the swelling joint in the OA rat model [52].

Our results showed that the most common combination of two CHMs was “Du-huo-ji-sheng-tang plus Shu-jiang-hou-xie-tang” in treating OA, followed by “Jia-wei-xiao-yao-san plus Du-huo-ji-sheng-tang” and “Jia-wei-xiao-yao-san plus Shu-jing-huo-zue-tang.” “Jia-wei-xiao-yao-san” (Dan-zhi-xiao-yao-san) was commonly used in menopausal female to ameliorate menopausal symptoms [53], so the menopausal women with OA were frequently prescribed with these two formulae.

The most commonly combination of three CHMs is “Du-huo-ji-sheng-tang, Gu-sui-pu plus Xu-duan”, followed by “Du-huo-ji-sheng-tang, Yan-hu-suo plus Nui-xi” and “Du-huo-ji-sheng-tang, Shu-jing-huo-zue-tang plus Yan-hu-suo.” “Gu-sui-pu” could inhibit the osteoclast activity in the OA mouse model [54] and prevent osteoporosis [55]. And “Yan-hu-suo” had anti-inflammatory effect with decreasing vascular permeability and restraining the development of adjuvant-induced edema in arthritic mice and rat models [56]. It also could alleviate pain with analgesic effect in a rat model [57].

TCM doctors in Taiwan frequently prescribe a combination of Chinese herbal formulae and Chinese single herb with the concepts of “pattern identification as the basis for determining treatment” and “sovereign, minister, assistant, and courier” [33,35,37]. Mostly, there were six items of CHM in a prescription for OA. Since female OA patients usually combined with menopausal syndrome and osteoporosis, the TCM doctors might combine use these CHM formulae more frequently in order to treat these two diseases.

There were some limitations in this study: (1) The diagnosis of OA among TCM doctors might mix with rheumatoid arthritis and other arthropathy or connective tissue disease, since the joint pain or arthralgia were similar. This might explain that the highest prevalence of OA in our study was 40-49 years, while OA is known to be a degenerative disease which commonly seen in elderly. (2) We only analyzed the utilization of CHM for treating OA, the utilization of acupuncture and Chinese tuina in treating OA were not included. (3) Since the therapeutic effectiveness of TCM for OA is not evaluated in this study, further efficacy studies will be executed in the future.

## Conclusions

This pharmaco-epidemiological study showed higher utilization of Chinese herbal medicine use of OA patients in the 40-49 age group and female patients, with Du-zhong (Eucommia bark) and Du-huo-ji-sheng-tang being the most commonly prescribed single herb and herbal formula, respectively.

The most common single herb and Chinese herbal formula are “Du-zhong” and Chinese “Du-huo-ji-sheng-tang.” The most common combination of two CHMs and triple CHMs are “Shu-jiang-hou-xie-tang plus Du-huo-ji-sheng-tang” and “Du-huo-ji-sheng-tang, Gu-sui-pu plus Xu-duan.” However, the therapeutic effects and safety of these CHM in treating OA still need to elucidate with well-defined randomized, double-blind, placebo-controlled clinical trials for further study.

## Abbreviations

CHM: Chinese herbal medicine; TCM: Traditional Chinese medicine; OA: Osteoarthritis; NHI: National Health Insurance; NHIRD: National Health Insurance Research Dataset; ICD-9-CM: International Classification of Diseases, Ninth Revision, Clinical Modification; WOMAC: Western Ontario and McMaster Universities Arthritis Index.

## Competing interests

The authors declare that they have no competing interests.

## Authors’ contributions

FPC and SJH conceived and designed the experiments. FPC, YCC and FJC performed the experiments. YCC and FJC analyzed the data. FJC and SJH contributed reagents/materials/analysis tools. FPC, CMC and SJH wrote the manuscript. FPC and SJH conceived the project. All authors read and approved the final manuscript.

## Pre-publication history

The pre-publication history for this paper can be accessed here:

http://www.biomedcentral.com/1472-6882/14/91/prepub
